# High-Dose Rifampicin Plus Albendazole Rapidly Clears Lymphatic Filariasis Circulating Filarial Antigen in a Randomised Clinical Trial: A Promising Step Toward Short-Course Macrofilaricidal Therapy

**DOI:** 10.3390/pathogens15020174

**Published:** 2026-02-05

**Authors:** Emmanuel Donawobuge Kutu, Derrick Adu Mensah, Vera Serwaa Opoku, John Boateng, John Opoku, Jubin Osei-Mensah, Charles Gyasi, Prince Obeng, Abu Abudu Rahamani, Monica Ahiadorme, Prince Dennis Atisu, Michael Agyemang Obeng, Eunice Kyaakyile Kuutiero, Nana Kwame Ayisi-Boateng, Derrick Boateng Kontoh, Sampson Twumasi-Ankrah, Linda Batsa Debrah, Alexander Yaw Debrah

**Affiliations:** 1 Department of Clinical Microbiology, School of Medical Sciences, Kwame Nkrumah University of Science and Technology, Private Mail Bag, University Post Office, Kumasi 00233, Ghana; donawusmotion@yahoo.com (E.D.K.); jhnboat2008@gmail.com (J.B.); abudulrahamani@yahoo.co.uk (A.A.R.); pkatisu135@gmail.com (P.D.A.); lindrousy@yahoo.com (L.B.D.); 2 Kumasi Centre for Collaborative Research in Tropical Medicine, Kwame Nkrumah University of Science and Technology, Private Mail Bag, University Post Office, Kumasi 00233, Ghana; derrickadumensah@yahoo.com (D.A.M.); vopoku88@gmail.com (V.S.O.); johnopoku93@gmail.com (J.O.); jubinom@yahoo.com (J.O.-M.); charles.gyasi19@gmail.com (C.G.); princeo@vt.edu (P.O.); mahiadorme@yahoo.com (M.A.); michaeloa75@gmail.com (M.A.O.); ekuutiero@gmail.com (E.K.K.); 3 Department of Theoretical and Applied Biology, Kwame Nkrumah University of Science and Technology, Private Mail Bag, University Post Office, Kumasi 00233, Ghana; 4 Department of Public Health, Akenten Appiah-Menka University of Skills Training and Entrepreneurial Development, Kumasi P.O. Box 1277, Ghana; 5 Department of Medical Laboratory Technology, Royal Ann College of Health, Kumasi P.O. Box KS 6253, Ghana; 6 German-West African Centre for Global Health and Pandemic Prevention (G-WAC), Partner Site Kumasi, Kwame Nkrumah University of Science and Technology, Private Mail Bag, University Post Office, Kumasi 00233, Ghana; 7 Department of Pathobiology, School of Veterinary Medicine, Kwame Nkrumah University of Science and Technology, Private Mail Bag, University Post Office, Kumasi 00233, Ghana; 8 Department of Basic Sciences, School of Basic and Biomedical Sciences, University of Health and Allied Sciences, Private Mail Bag 31, Ho, Volta Region, Ghana; 9 Department of Medicine, School of Medical Sciences, Kwame Nkrumah University of Science and Technology, Private Mail Bag, University Post Office, Kumasi 00233, Ghana; ayisi31@gmail.com; 10 University Hospital, Kwame Nkrumah University of Science and Technology, Private Mail Bag, University Post Office, Kumasi 00233, Ghana; 11 Department of Pharmaceutics, Faculty of Pharmacy and Pharmaceutical Sciences, College of Health Sciences, Kwame Nkrumah University of Science and Technology, Private Mail Bag, University Post Office, Kumasi 00233, Ghana; kontohderrick4@gmail.com; 12 Department of Statistics and Actuarial Science, Faculty of Physical and Computational Sciences, College of Science, Kwame Nkrumah University of Science and Technology, Private Mail Bag, University Post Office, Kumasi 00233, Ghana; sampson.ankrah@yahoo.com; 13 Department of Medical Diagnostics, Kwame Nkrumah University of Science and Technology, Private Mail Bag, University Post Office, Kumasi 00233, Ghana

**Keywords:** lymphatic filariasis, high dose, rifampicin, albendazole, circulating filarial antigen, macrofilaricidal

## Abstract

**Background**: The lack of a short-course of safe and effective macrofilaricidal therapy for lymphatic filariasis (LF) hinders elimination efforts, especially in the endgame scenario. Preclinical studies in mice demonstrated that high-dose rifampicin (RIF) plus albendazole (ALB) produced macrofilaricidal effects within seven days, prompting this randomised, open-label, parallel-group, interventional phase II pilot trial to determine the efficacy of high-dose RIF plus ALB against LF in humans. **Methods**: In three LF-endemic districts of Ghana’s Upper East Region, circulating filarial antigen (CFA)-positive individuals aged 18 to 55 years identified using the Alere Filariasis Test Strip were enrolled into the study. The participants were randomised through a centralized computer-generated randomisation into four treatment arms. They were treated according to the arm they were assigned to and followed up at 4-, 6-, 12-, and 18-months post-treatment to monitor changes in CFA status and levels, as well as adverse events. Outcome assessors were blinded to minimize assessment bias. **Results**: A total of 69 eligible participants were randomised into four treatment arms: RIF (35 mg/kg/day) + ALB (400 mg/day) for 7 days (*n* = 17), RIF (35 mg/kg/day) + ALB (400 mg/day) for 14 days (*n* = 18), ALB alone for 14 days (*n* = 17), and an untreated controlled group participating in standard mass drug administration (*n* = 17). All regimens were well tolerated, with no serious adverse events. Even though CFA positivity declined across all groups, with maximal reductions at 18 months, the RIF + ALB 7-day regimen consistently showed the highest decline, while ALB alone was the least effective. RIF + ALB groups exhibited early antigen decline by 4 months, unlike comparator groups, where reductions occurred from 12 months. **Conclusions**: These findings suggest macrofilaricidal activity of high-dose RIF plus ALB, supporting further trials in larger, microfilaraemic populations. The trial was registered in the Pan African Clinical Trials Registry on 9 September 2020 under the code PACTR202009704006025.Funding was by the European and Developing Countries Clinical Trials Partnership 2 (EDCTP2), with grant code TMA2018SF-2451-ASTAWOL, and by the German Federal Ministry of Education and Research (Bundesministerium fur Bildung und Forschung-BMBF) under agreement with Gesellschaft für Internationale Zusammenarbeit (GIZ) through agreement number: 81204851.

## 1. Introduction

Lymphatic filariasis (LF) is an ancient yet persistent neglected tropical disease [1] whose chronic manifestations including lymphoedema, elephantiasis, and hydrocele continue to cause lifelong disability, poverty, social stigma, and profound social and economic consequences in the 21st century [2,3].

The disease is diagnosed based on parasitological detection of microfilariae (MF), antigen detection, and antibody detection [4]. Although MF detection is an established method, it is limited by low sensitivity and the nocturnal periodicity of MF makes blood collection challenging [4]. Consequently, antigen-based immunoassays such as the Og4C3 TropBio enzyme-linked immunosorbent assay (ELISA) and the filariasis test strip (FTS) are now widely used for LF diagnosis and surveillance [5]. Both the FTS and the Og4C3 TropBio ELISA demonstrate up to 100% sensitivity and over 98% specificity in highly microfilaraemic populations. However, the sensitivity of both tests drops to <73% when MF density is <1 MF/mL, and in post mass drug administration (MDA) settings [6,7]. Both assays detect *W. bancrofti* circulating filarial antigen (Wb-CFA/Wb123) using monoclonal antibodies, but differ in application: the Og4C3 TropBio ELISA provides quantitative CFA measurements in serum, plasma, or hydrocele fluid, enabling sensitive detection and monitoring of treatment efficacy, whereas the FTS is a rapid, qualitative, point-of-care test based on the AD12 monoclonal antibody and is better suited for large-scale mapping and post-treatment surveillance [5,6,8,9].

The biggest campaign against LF has been the Global Programme to Eliminate Lymphatic Filariasis (GPELF), which aims to eliminate the disease through MDA of anti-parasitic drugs using ivermectin (IVM), diethylcarbamazine (DEC), and albendazole (ALB) to at-risk populations [10]. IVM causes paralysis and immune-mediated clearance of worms via glutamate-gated chloride channel activation, and ALB works by disrupting microtubule-dependent nutrient uptake [11]. A major limitation of the drugs used in MDA is that, while IVM and ALB are highly effective against microfilariae, they have little or no direct activity against adult *W. bancrofti*. Consequently, adult worms can continue to produce microfilariae, necessitating repeated treatment rounds and enabling recrudescence following MDA cessation [12]. Additionally, global mobility poses a risk of reintroducing LF into areas where it has been eliminated, while emerging signs of suboptimal efficacy of IVM raise concerns since there are no alternative treatments suitable for MDA [13,14]. Therefore, developing and implementing new drugs or improved regimens effective against the adult worms is essential to accelerate elimination efforts in the remaining endemic regions and foci and enhance cost-effectiveness by reducing unnecessary treatment of uninfected individuals within MDA programs.

The challenge in the search for macrofilaricidal treatment for LF is one that can eliminate the infection in a short treatment time frame, ideally 7 days or less [15].

*Wolbachia* endosymbiotic bacteria are present in most filarial worms and are crucial for their fertility and survival [16]. Studies have shown that antibiotics such as tetracycline and its derivatives, which are effective against *Wolbachia*, can lead to several detrimental effects on the host nematode. These effects include the inhibition of embryogenesis, disruption of MF production, and prevention of the development from infective larvae (L3) to adult stages [17]. For instance, doxycycline therapy has been shown to lead to *Wolbachia* depletion and subsequently lead to macrofilaricidal effects such as long-term sterility in the adult worms when administered for 4–6 weeks [18,19]. However, in order to achieve significant and sustained >90% *Wolbachia* depletion and macrofilaricidal effect, long-term treatment is required, making doxycycline unsuitable for MDA programs [15]. It is also contraindicated in children and pregnant and lactating women [20].

Rifampicin (RIF) has demonstrated superior anti-*Wolbachia* efficacy compared to tetracycline-class antibiotics in both in vitro and in vivo studies. As a matter of fact, a preclinical mouse model research [20] showed that RIF is about 16 times more potent at depleting *Wolbachia* compared to doxycycline. However, clinical trials did not replicate these preclinical successes when RIF was administered at standard doses (10 mg/kg) in LF patients [21,22]. Results from mouse model research and simulation studies [15] suggest that a 30–40 mg/kg dose of RIF is required in humans to achieve drug exposures that could lead to the elimination of *Wolbachia* and a macrofilaricidal effect comparable to the macrofilaricidal effects seen with doxycycline treatment. Turner and colleagues found that a combination of high-dose RIF (35 mg/kg) and ALB in a mouse model resulted in 99% *Wolbachia* depletion, halted embryogenesis, halted MF production, and had a significant macrofilaricidal effect against the human filarial *Brugia malayi* in 7 days of treatment and *Onchocerca ochengi* in 14 days of treatment [15]. Based on these promising results, they recommended an urgent clinical trial to evaluate whether this short-course regimen can replicate the macrofilaricidal efficacy of doxycycline in humans. Encouragingly, studies showed that increasing rifampicin doses up to fourfold is safe for one-month periods in patients with tuberculosis [23]. Hence, this study sought to test the efficacy (reduction in circulating filarial antigen levels) of the combination of high-dose RIF and ALB compared to treatment with ALB alone and ‘no treatment’ (other than ivermectin) against LF using the filariasis test strip (FTS). The specific objectives included (1) assessment of the adverse events related to combination of RIF and ALB in the treatment of LF, (2) assessment of modulation in CFA levels as a measure of the presence of adult worms through the use of the filarial test strip at 18 months after treatment onset and (3) assessment of the efficacy (reduction in CFA levels) of the combination of rifampicin plus albendazole compared to treatment with albendazole only and ‘no treatment’ (other than ivermectin) against lymphatic filariasis using the Og4C3 antigen test 18 months after treatment onset.

## 2. Materials and Methods

### 2.1. Trial Area Description

**Figure 1:**
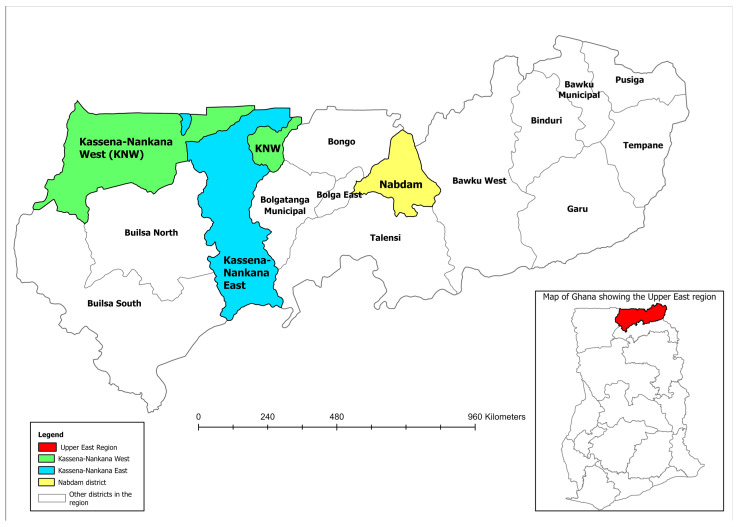
Location of the study districts within the Upper East Region of Ghana, with national context shown in the inset map. (Created by the authors using Environmental Systems Research Institute’s Geographic Information System software, ArcGIS Pro version 3.1.0).

The study was conducted in 64 LF-endemic communities within 12 sub-districts across the Kassena-Nankana East Municipal, the Kassena-Nankana West and the Nabdam Districts, located in the Upper East Region of Ghana (Figure 1). These districts are situated within the Guinea Savannah ecological zone and are labelled as some of the LF “hotspot” districts in Ghana [9,24]. The population is predominantly rural, with livelihoods based on subsistence agriculture and animal rearing. The study area has distinct wet and dry seasons. The rainy season extends from May to October, while the dry season spans from October to April. The mean annual rainfall across the study area is approximately 950 mm, and there are numerous small dams and dugouts, including the Tono irrigation dam in Navrongo, which provides water for year-round irrigation and also creates favourable breeding sites for mosquitoes [25].

### 2.2. Trial Design, Study Population, Sample Size, and Eligibility Criteria

This was a prospective, randomised, controlled, monocentric, open-label, parallel-group, interventional phase II pilot trial. The trial was registered in the Pan African Clinical Trials Registry under the code PACTR202009704006025 https://pactr.samrc.ac.za/Search.aspx, registered on 9 September 2020. The study was conducted among LF-infected individuals living in the Kassena-Nankana East Municipal, Kassena Nankana West District, and Nabdam District in the Upper East Region of Ghana. Persons with LF infection as detected by the FTS test who met the inclusion/exclusion criteria were recruited to participate in this clinical trial.

A total of 69 participants were recruited for this clinical trial. This was a non-confirmatory pilot study to gain first experience regarding the primary and secondary endpoints under the intended treatment regimens; hence, the sample size was not justified by a statistical argument but based on the experience from previous trials. According to Julius, a minimum of 12 participants per treatment arm is recommended in pilot trials to generate preliminary estimates of treatment effects [26].

Eligible individuals were adults aged 18–55 years, body weight >45 kg, CFA-positive (with or without Mf), in good health, and without a history of tuberculosis. Exclusion criteria were people with known hypersensitivity to the investigational drug, acute/chronic hepatitis, medical conditions posing risks or affecting trial results, substance abuse, history of neurological, cardiac, or pulmonary diseases, anaemia, abnormal hepatic and renal enzymes [Alanine Aminotransferase (0–44 U/L), Aspartate Aminotransferase (0–40 U/L), Gamma-Glutamyl Transferase (0–55 U/L), Creatinine (55–124 µmol/L)] assessed by biochemistry analysis using the validated Chemistry analyser (Selectra Pro S, ELITechGroup Inc., Dieren, The Netherlands). Pregnant/breastfeeding women and females unwilling to use effective contraception were also excluded.

### 2.3. Recruitment, Randomisation, and Treatment of Trial Participants

Screening and enrolment took place directly within the study communities. With the help of community health volunteers, community members were informed to meet the study team at a suitable location chosen by the community opinion leaders. At the meeting, the study procedures were thoroughly explained to the potential participants, and all their questions were answered satisfactorily. Interested volunteers gave their consent by signing or thumb-printing the informed consent form. Participants were tested for CFA on-site, and those who tested positive were recruited per the eligibility criteria. Recruitment and enrolment took place from 7 May to 15 July 2022 while treatment took place from 15 July to 4 August 2022. The follow-ups were as below:

- 4-month follow-up: 3–8 November 2022.
- 6-month follow-up: 25–27 January 2023.
- 12-month follow-up: 20–25 July 2023.
- 18-month follow-up: 26–31 January 2024.

A total of 69 FTS/CFA positive participants who had taken/consumed different rounds of MDA were enrolled and randomised into four treatment arms, through a central randomisation carried out according to a predefined randomisation list which comprised treatment codes from 1 to 69 and the name of the respective treatment for each code. When a participant was found eligible for treatment, his/her identification (ID) was sent to the data management team at KNUST, and the next treatment number in the list was assigned to the participant. Randomised participants either received rifampicin (35 mg/kg/day) plus albendazole (400 mg/day) for 7 or 14 days, or albendazole alone (400 mg/day) for 14 days, or they received no therapy but took part in the standard community MDA. Participants were treated in their communities under directly observed treatment (DOT) by the study trial clinician, pharmacist, and the research team. All study participants were followed-up at 4-, 6-, 12- and 18-months post-treatment. At the 6-month follow-up time point, all participants were given the standard MDA drugs for ethical reasons and standard of care.

Participants and the study team were not blinded to the treatment allocations. However, outcome assessors conducting MF and Og4C3 TropBio enzyme-linked immunosorbent assay (ELISA) analyses (as explained in the laboratory examinations) were blinded to minimize assessment bias.

The treatment arms were as follows:

- Treatment Arm 1 (TA1): Rifampicin 35 mg/kg/day plus albendazole 400 mg/day for 7 days = 17 participants
- Treatment Arm 2 (TA2): Rifampicin 35 mg/kg/day plus albendazole 400 mg/day for 14 days = 18 participants
- Treatment Arm 3 (TA3): Albendazole 400 mg/day for 14 days = 17 participants
- Treatment Arm 4 (TA4): No treatment (control) = 17 participants

### 2.4. Field Examinations

Field examinations performed in this trial included obtaining data on participants’ demographics and past MDA records, assessment of participants’ clinical history, and a test for circulating filarial antigen (CFA) status.

#### Assessment of Circulating Filarial Antigen Status

Assessment of CFA status was done on site at the communities by the use of the Alere filariasis test strips (Abbott Diagnostics Scarborough, Inc., Scarborough, ME, USA) according to the manufacturer’s instructions. Briefly, 75 µL of the whole blood sample collected through finger-prick by the use of a sterile disposable lancet was added to the sample application pad of the FTS, and results were read at 10 min by two independent readers. Tests were interpreted as positive for CFA when both the test and control areas showed pink bands and negative when only the control band appeared. The FTS results were semi-quantified using the technique by Chesnais, and Mensah and colleagues [5,9], which used the intensity of the test band to grade the results into the following: grade 0 = negative, grade 1 = test band produces lower intensity than control band, grade 2 = test band produces equal intensity to control band, and grade 3 = test band produces higher intensity than control band. Tests with no band at the control area were interpreted as invalid.

### 2.5. Laboratory Examinations

#### 2.5.1. Quantification of Circulating Filarial Antigen

CFA levels were also measured using the Og4C3 TropBio ELISA (Cellabs, Sydney, NSW, Australia) filariasis test. This test was carried out at the Immuno-parasitology laboratory, at the Department of Medical Diagnostics, KNUST, Ghana, and it was done according to the manufacturer’s instructions. For this test, 3.5 mL of whole blood collected in ethylenediaminetetraacetic acid (EDTA) tubes was processed in the field laboratory (War Memorial Hospital, Navrongo, Ghana) by centrifuging the blood at 2000× *g* for 10 min, and 1 mL of plasma was preserved in liquid nitrogen and finally transported to KCCR, KNUST and stored at −20 °C for Og4C3 antigen analysis. Briefly, the test involved bringing plasma samples, microtitre plates, and all reagents to room temperature before analysis. For each sample, 70 µL of plasma was diluted in 210 µL of sample diluent, vortexed, heated at 100 °C for 5 min, and centrifuged at 10,000× *g* for 5 min. Then 50 µL of the supernatant was added to a 96 microtitre plate. Samples were run in duplicates alongside standard antigens and a control. The plate was incubated at 37 °C for 60 min, washed, and treated with anti-Onchocerca antibody and incubated at 37 °C for 45 min, followed by anti-rabbit horseradish peroxidase (HRP) conjugate plus another incubation at 37 °C for 45 min. After washing, tetramethylbenzidine (TMB) substrate was added and incubated for 15 min in the dark, and the reaction was stopped with a stopping solution. Absorbance was read at 450 nm and 620 nm using a SpectraMax Plus 384 Microplate Reader (GMI Trusted Laboratory Solutions, Ramsey, MN, USA).

Result interpretation was carried out according to the manufacturer’s protocol as follows: the optical density (OD) of standard 2 was used as the cut-off if within ±10% of 0.35 or, a default value of 0.35 was used if standard 2 OD fell outside the range and interpreted (Negative = any OD below the set cut-off; Low Positive = any OD from the cut-off but below 0.8; Medium Positive = OD from 0.8–1.0, and High Positive = OD > 1.0).

#### 2.5.2. Determination of Microfilariae Load

Microfilaraemia assessments were carried out using the Giemsa filtration technique as previously described [9]. The test was performed in the field laboratory at the War Memorial Hospital, Navrongo, in the Upper East Region of Ghana. Briefly, MF levels were determined from a 10 mL whole blood sample collected between 21:00 and 23:00 due to the nocturnal periodicity of *W. bancrofti* [27]. To isolate MF, 1 mL of the blood was passed through a Whatman Nucleopore filter (5 µm). The filter was then stained using the Giemsa method, and MF were counted under a light microscope.

#### 2.5.3. Assessment of Drug Safety

All participants were actively monitored and assessed by the trial clinician during and after administration of study medications for the occurrence of adverse events (AEs). All study participants, regardless of the treatment arm they were assigned to, were asked to come for the daily treatment visits for at least 14 days for safety assessment where the trial clinician asked for adverse events and performed physical examination or other diagnostic procedures as necessary. An adverse event was defined as any unfavourable and unintended sign (including abnormal laboratory findings), symptom, or disease temporally associated with the medical products used in the study. A serious adverse event was defined as any untoward medical occurrence that at any dose results in death, life-threatening condition, requirement for inpatient hospitalization or prolongation of existing hospitalization, persistent or significant disability/incapacity, or congenital anomaly/birth defect. In addition to the first evaluation of an adverse event that is performed by the trial clinician, a second evaluation with respect to seriousness, causality, and expectedness was performed. All study procedures were conducted in accordance with good clinical practice (GCP) guidelines to ensure that the safety and rights of participants were safeguarded. Participants were followed up at 4-, 6-, 12- and 18-months post-treatment to assess treatment efficacy. At the 6-month follow-up, ivermectin and albendazole were administered to all participants, including those in the no-treatment arm. This was done in accordance with ethical considerations and the standard of care for the LF infection.

### 2.6. Data and Statistical Analysis

All trial data were entered in REDCap (Research Electronic Data Capture) [28,29] during the trial using double data entry on site in Ghana. The REDCap was hosted at the Department of Statistics, KNUST, Ghana. All statistical analyses were performed using Stata version 16 (Stata Corporation LLC, College Station, TX, USA) and SAS version 9.4 (SAS Institute Inc., Cary, NC, USA). Data visualisation was performed using GraphPad Prism 10.2 (GraphPad Software, Inc., San Diego, CA, USA).

Two datasets, the per protocol (PP) and intention-to-treat (ITT), were used in analysing treatment efficacy. The PP refers to participants who completed treatment per the protocol, while the ITT refers to participants who took the drugs at least once. Subjects dropping out of the trial prior to randomisation were listed as screen failures including the reasons for dropping out. Subjects dropping out of the trial after randomisation and also during treatment were analysed using all available data (ITT analysis). Subjects missing more than 3 consecutive treatment days were analysed using all available data (ITT analysis). All available data from the participants who dropped out were used for the ITT analysis. Given that the study was a non-confirmatory pilot trial, efficacy analysis primarily relied on the PP dataset, which was for participants who completed treatment per the protocol and were present at 4-, 6-, 12- and 18-months post-treatment and had taken IVM at the 6-month follow-up. The ITT dataset was used to describe baseline characteristics, assess adverse events, and corroborate findings from the PP analyses. Continuous variables were assessed for normality using the Shapiro–Wilk test. Baseline demographic characteristics were compared among the treatment groups using Fisher’s exact test, analysis of variance (ANOVA) test and Kruskal–Wallis test. Proportions of CFA-positive and CFA-negative individuals at each time-point were compared among the groups using Fisher’s exact tests, while proportions of CFA-positive and CFA-negative before and after treatment in each group were compared using the McNemar–Bowker test. The Kruskal–Wallis equality-of-populations rank test was used to compare CFA levels between treatment arms at each time-point. Changes in the levels of CFA were calculated as percentages from both the geometric mean and median of the baseline data and analysed within each group using the Wilcoxon signed-rank test and Dunn’s test. *p*-values < 0.05 were considered statistically significant at 95% confidence interval.

## 3. Results

A total of 730 volunteers from 64 communities across 12 sub-districts in the three study districts were screened for *W. bancrofti* CFA using the FTS test. This group included 407 females and 323 males, with a mean age of 38.60 years (SD = 10.00). The majority of the participants (43.3%, *n* = 316) were from the Kassena Nankana West District. Most of those screened (85.9%, *n* = 627) tested negative for CFA. Among the 14.1% (*n* = 103) who tested positive for CFA, the majority (71.8%, *n* = 74) were of FTS grade 1, while only one participant (1.0%) was FTS grade 3. None of the participants tested positive for MF.

**Figure 2:**
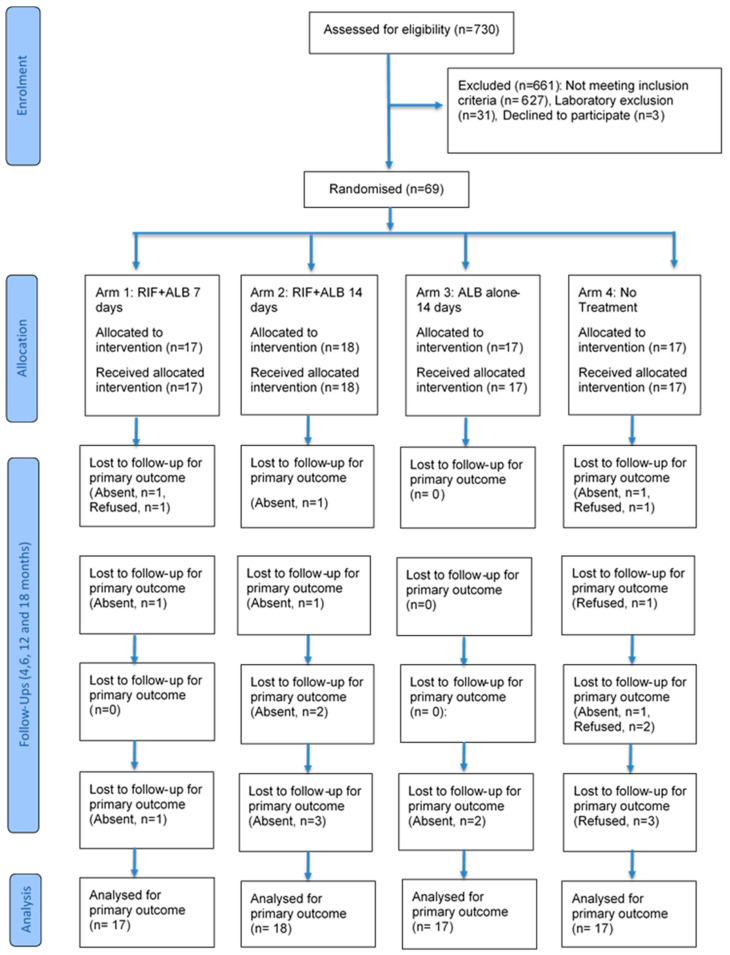
Participant flow chart, showing the number of participants screened, randomised, treated, followed up, and analysed.

A total of 69 CFA-positive participants, as determined by the FTS test, were enrolled for the clinical trial and randomised into four treatment arms. The participants were treated correspondingly with the dose of the treatment arm they were randomised into, and they were followed up at 4, 6, 12, and 18 months after treatment. Below is a flowchart detailing participants’ recruitment, treatment, and follow-up (Figure 2).

### 3.1. Safety Assessments

No serious adverse events (SAEs) were recorded in the trial. However, a total of 18 AEs types were experienced by 40 (58.0%) participants during treatment, with the majority of the reports being reddish discolouration of urine, which occurred significantly in TA1 and TA2. Headache was the only AE recorded in all the treatment arms (Table 1 and Appendix A, Table A1). Participants were given medications for the AEs they experienced. These medications included diclofenac tablet, diclofenac gel, paracetamol, cetrizine, Nebilet, cloxacillin, artemether plus lumefanthrine and Lufart.

### 3.2. Baseline Characteristics of Study Participants

The majority (43.5%, *n* = 30) were from the Nabdam district. Females constituted 52.2% of the 69 participants enrolled into the study. While only four participants (5.8%) had never received MDA before, the majority (53.6%) had completed five or more rounds. There were no differences statistically in the distribution across treatment arms in terms of gender, district, or the number of MDA rounds received. Similarly, the distribution of different FTS grades/scores across treatment groups showed no statistical difference. Notably, 34.8% (*n* = 24) of the enrolled FTS-CFA positive participants tested positive for CFA per the TropBio ELISA test, and the distribution of these 24 Og4C3 CFA positive participants among the treatment arms was not different. Table 2 provides an overview of the participants’ demographic and clinical features (Table 2).

### 3.3. Changes in CFA Status Across the Study Time-Points Within the Various Treatment Groups

All the four treatment arms had a reduction in the number of CFA-positive cases per the FTS test (FTS/CFA+) at all the follow-up time-points compared to the baseline. The highest rate of seroreversion (change in status from FTS/CFA positive to FTS/CFA negative) occurred at the 18-month post-treatment follow-up, and it was so for all the treatment arms. The rate of seroreversion did not differ statistically across the treatment arms at any of the follow-up time points. However, treatment arm 1 (RIF + ALB-7 Days) consistently showed the highest rate of seroreversion across all follow-ups. Conversely, treatment arm 3 (ALB alone-14 Days) recorded the lowest rate of seroreversion across all follow-ups (Table 3).

Using the Og4C3 TropBio ELISA test, similar findings to the FTS/CFA results were observed, where all treatment arms showed a decline in the number of Og4C3/CFA positive cases across all follow-up time points, with no significant difference in seroreversion rates between the treatment arms at any follow-up time point. However, in treatment arm 2 (RIF + ALB for 14 days), all participants who were Og4C3/CFA positive at baseline and present for follow-up at 18-month post-treatment had seroreverted, a significant change from baseline (*p* = 0.031). Table 3 shows CFA status changes that occurred in the various treatment arms from baseline through all the follow-up timepoints. Similar statistical observations were obtained when the analyses were carried out per protocol (Table 3) or per intention-to-treat (Appendix A, Table A2).

### 3.4. Changes in FTS Grades/Scores Across the Study, Time-Points in the Various Treatment Arms

**Figure A1:**
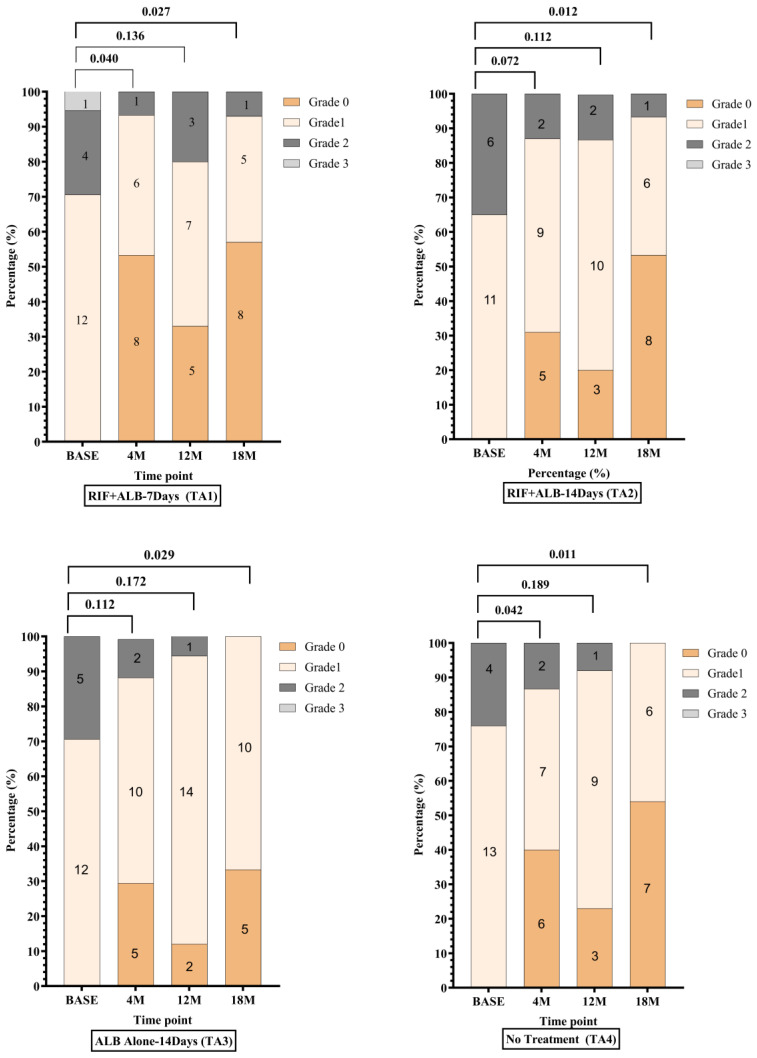
(PP) Changes in FTS grades/scores across the study time-points in the various treatment arms. McNemar–Bowker test for comparison was performed using baseline FTS grades/scores as comparator for participants who were present at each follow-up. Level of significance, *p*-value < 0.05. Only participants who were treated per protocol (PP) and had taken IVM at 6 months were included in analysis for 12- and 18-month follow ups. TA-Treatment Arm, RIF-Rifampicin, ALB-Albendazole, BASE-Baseline, M-Month.

**Figure A2:**
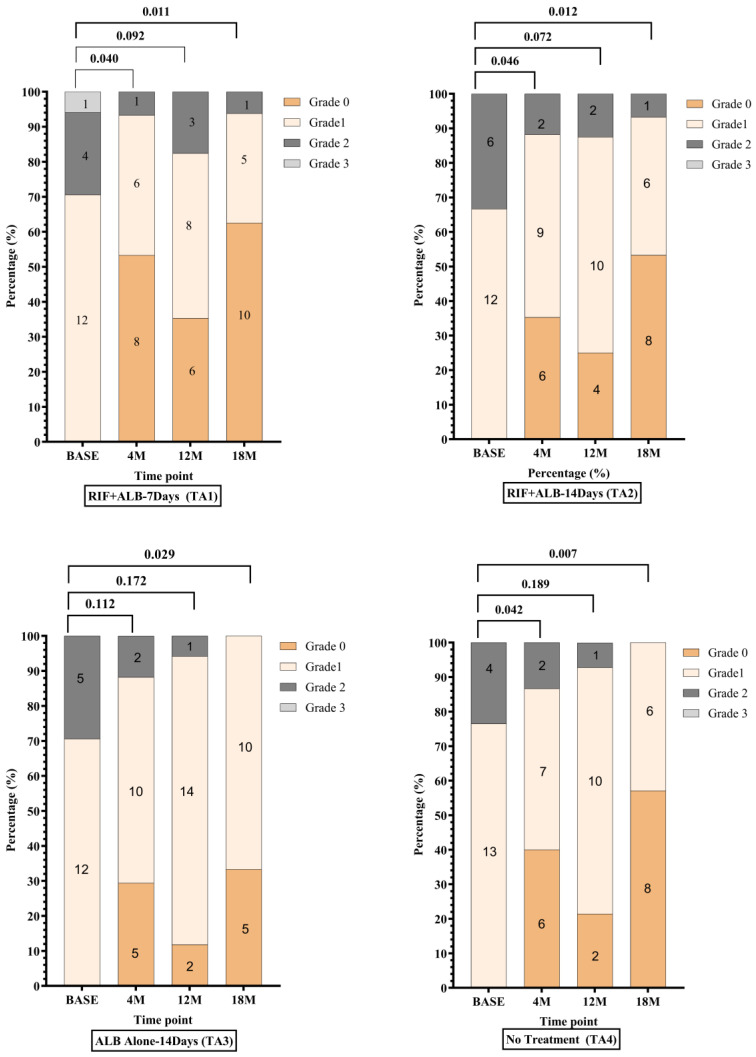
ITT changes in FTS grades across the study time-points in the various treatment arms. McNemar–Bowker test for comparison was performed using baseline FTS scores as comparator for participants who were present at each follow-up. Level of significance, *p*-value < 0.05. Abbreviations: TA: Treatment Arm, BASE: Baseline, M: Month, RIF: Rifampicin, ALB: Albendazole.

The McNemar–Bowker test was performed using baseline FTS grading as the reference point to compare with the FTS grades/scores at the different follow-ups in the various treatment arms. At 4-month follow-up, a significant difference in FTS grading (*p* < 0.05) was observed in all the treatment arms except for the “ALB-alone-14 Days” treatment arm. But at the 12-month follow-up, FTS grades/scores in all treatment arms did not differ from that at baseline. However, by the 18-month follow-up, a significant difference in FTS grading was noted across all treatment arms when compared to baseline. Similar statistical observations were recorded for the Per Protocol (PP) Analyses (Appendix A, Figure A1) and Intention-to-Treat (ITT) Analyses (Appendix A, Figure A2).

### 3.5. Changes in Og4C3 Antigen Levels Across the Study Time-Points

**Figure 3:**
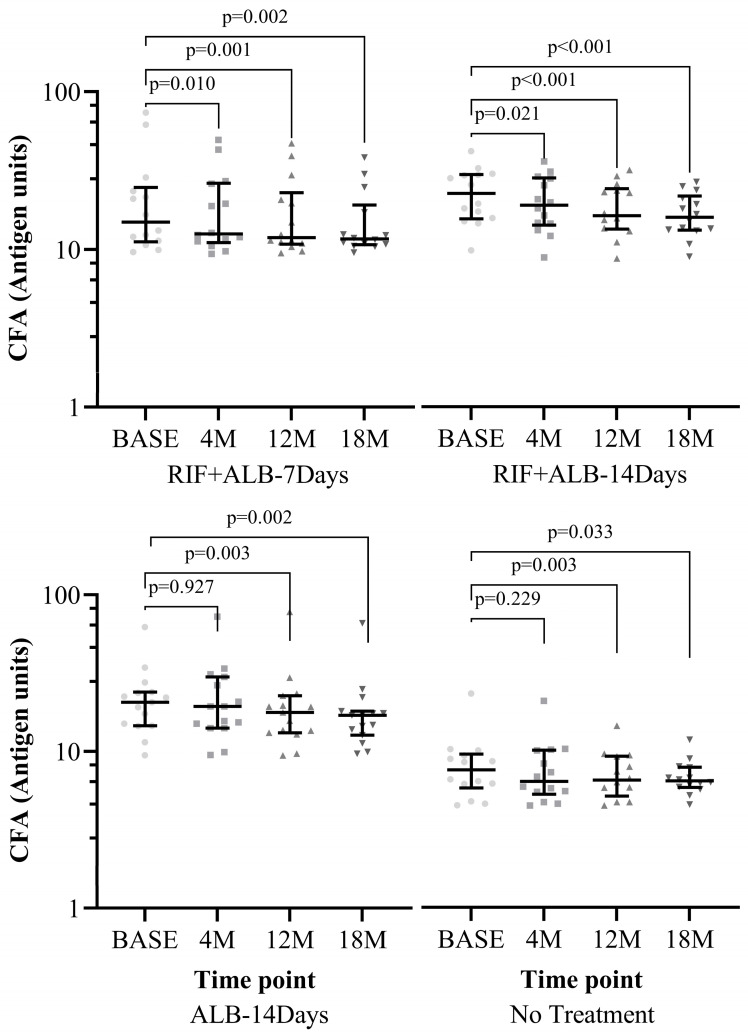
(PP) Comparison of Og4C3 circulating filarial antigen (CFA) levels between baseline and each follow-up point within each treatment arm. Antigen unit axis expressed in log10 scale. Wilcoxon signed-rank test used for all treatment arms except “RIF + ALB-14 Days” arm for which Dunn’s test was used. Only participants who were treated per protocol (PP) and had taken IVM at 6 months were included in analysis for 12- and 18- month follow ups. Level of significance set at *p*-value < 0.05. Abbreviations: BASE = Baseline, M: Month, RIF: Rifampicin, ALB: Albendazole.

**Figure A3:**
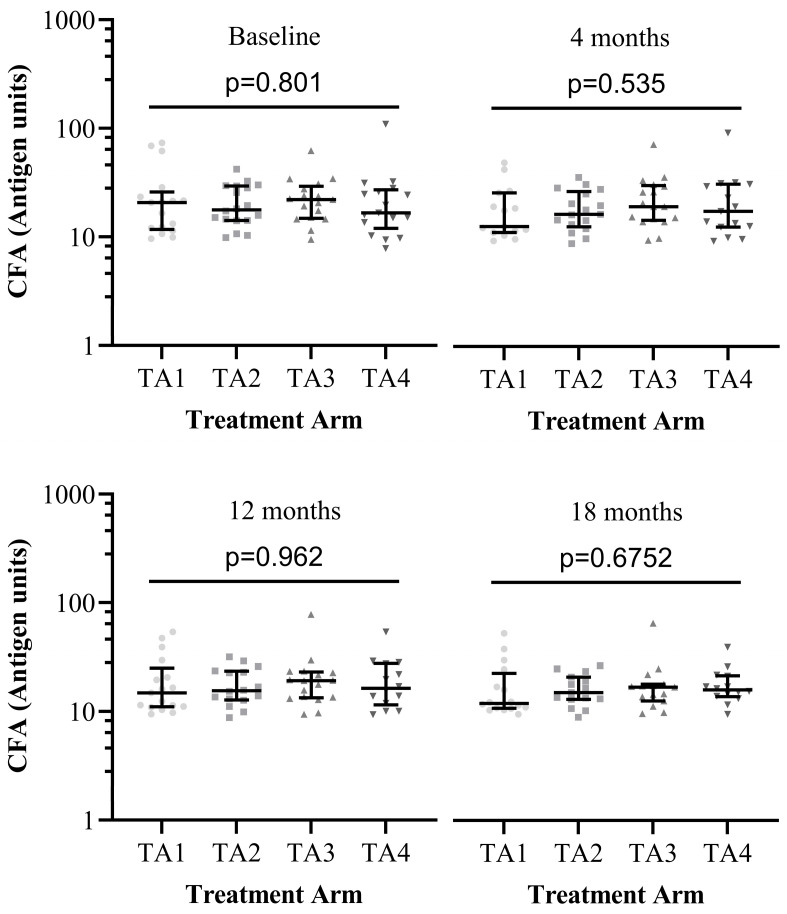
PP comparison of Og4C3 circulating filarial antigen (CFA) levels between treatment arms at each study time-point. Antigen unit axis expressed in log10 scale. Kruskal–Wallis equality-of-populations rank test used to compare CFA levels between treatment arms at each time-point. Level of significance, *p*-value < 0.05. Only participants who were treated per protocol (PP) and had taken IVM at 6 months were included in analysis for 12- and 18-month follow ups. Abbreviations: TA-Treatment Arm, TA1 = Rifampicin (RIF) +Albendazole (ALB)-7 Days; TA2 = RIF + ALB-14 Days, TA3 = ALB alone-14 Days, TA4 = no treatment.

**Figure A4:**
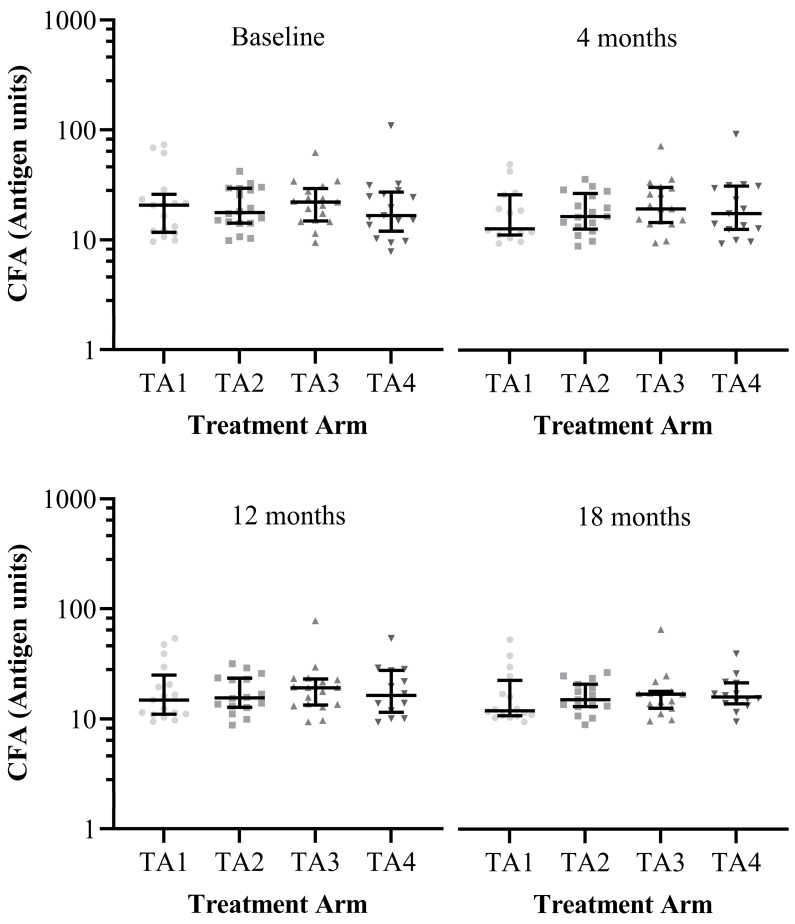
ITT comparison of Og4C3 CFA levels among the treatment arms across study time-points. Antigen unit axis expressed in log10 scale. Kruskal–Wallis equality-of-populations rank test used to compare CFA levels among the treatment arms at each time-point. Level of significance, *p*-value < 0.05. TA-Treatment Arm, TA1 = RIF + ALB-7Days; TA2 = RIF + ALB-14 Days, TA3 = ALB alone-14 Days, TA4 = no treatment.

**Figure A5:**
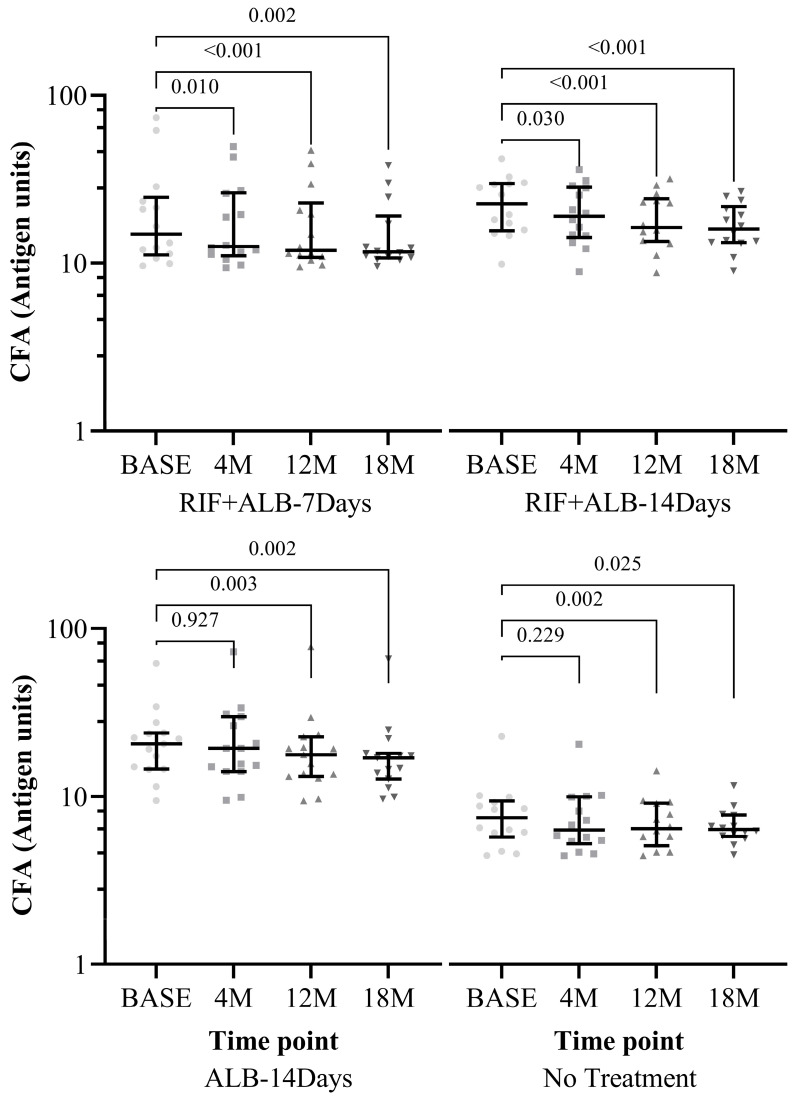
ITT comparison of Og4C3 circulating filarial antigen (CFA) levels across study time-points for each treatment arm. Antigen unit axis expressed in log10 scale. Wilcoxon signed-rank test used for all treatment arms except “RIF + ALB-14 Days” arm, for which ANOVA with Dunn’s post hoc test was used. Level of significance, *p*-value < 0.05. Abbreviations: BASE = Baseline, M: Month, RIF: Rifampicin, ALB: Albendazole.

The Levels of Og4C3 antigen units were measured using the Og4C3 TropBio ELISA test. When the levels of antigen units were compared among the treatment arms, no significant difference was observed between the treatment arms, either at baseline or at any of the follow-up time-points for the PP analyses (Appendix A, Figure A3) or the ITT analyses (Appendix A, Figure A4). Subsequently, within each treatment arm, the antigen levels at each follow-up time-point were compared to the antigen levels at baseline. As shown in Figure 3 below (for the PP analyses), the results of the “RIF + ALB-7 Days” and “RIF + ALB-14 Days” treatment arms showed a significant reduction in antigen levels as early as 4-months post-treatment and continued till 18 months. In contrast, the “ALB-alone-14 Days” and the “no treatment” arms only started to achieve significant reduction in antigen levels at 12-months post-treatment. This was also the case when the analyses were carried out per ITT (Appendix A, Figure A5).

### 3.6. Association Between FTS Test and Og4C3 TropBio ELISA Test Across Study Time-Points

Out of the 69 FTS/CFA positive participants recruited at baseline, 24 (34.8%) tested positive for the Og4C3/CFA by the TropBio ELISA test. It was observed that the percentage agreement between the two tests decreased with increasing follow-up time. While there was 43.6% agreement at the 4-month follow-up, the percentage decreased to 28.6% at the 12-month follow-up and further decreased to 24.1% at the 18-month follow-up. However, there was a significant association between FTS/CFA positivity and Og4C3/CFA positivity across all follow-ups. Notably, except at the 4-month follow-up where 3 (12.0%) of the FTS/CFA negative participants tested positive for Og4C3 CFA, all FTS/CFA negatives consistently tested negative for Og4C3 CFA during the other follow-ups (Table 4).

### 3.7. Association Between FTS/CFA Grades/Scores and Levels of Og4C3 Antigen Units

**Figure 4:**
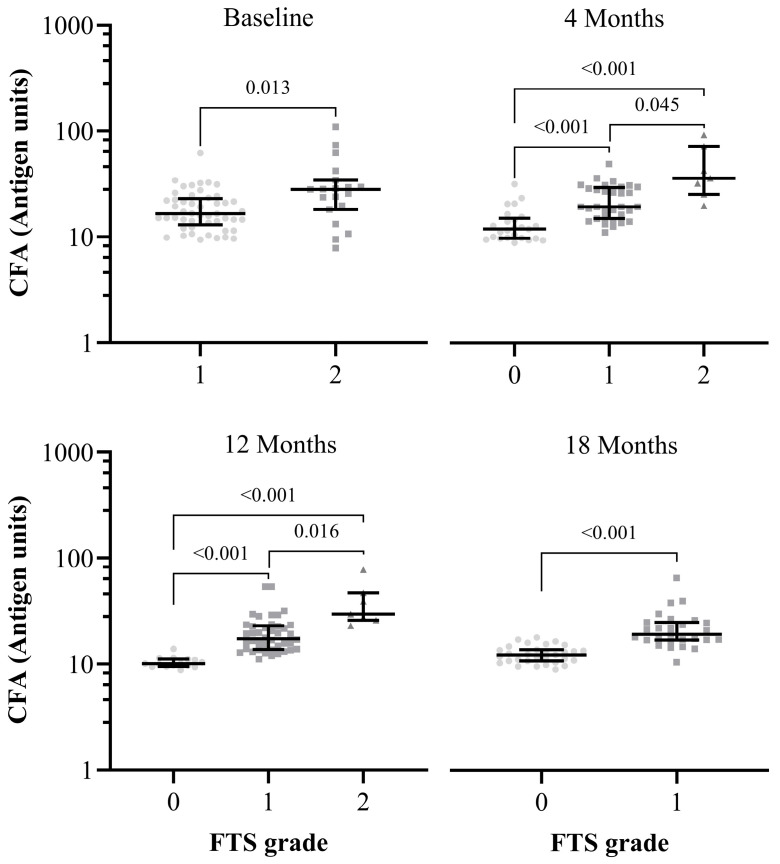
(ITT) Association between FTS grades/scores and Og4C3 CFA levels. Mann–Whitney test was used for baseline and 18-month follow-up. while Kruskal–Wallis test was used for 4- and 12-month follow-ups. FTS grade 3 was excluded from the baseline analysis, and FTS grade 2 was excluded from the 18-month follow-up analysis due to the small number of participants in these categories. Abbreviations: FTS—Filariasis Test Strip, CFA—Circulating Filarial Antigen.

Significant associations were observed between FTS/CFA grades/scores and Og4C3 antigen units. Higher FTS/CFA grades/scores were significantly (*p* < 0.05) associated with higher Og4C3 antigen units (Figure 4).

## 4. Discussion

The “holy grail” of LF treatment is a macrofilaricidal therapy that can eliminate infection within a short course, ideally no longer than seven days [15]. Our study is the first concerted clinical trial that demonstrates accelerated clearance of *W. bancrofti* CFA by the combination of high-dose RIF and ALB in LF infection, marking a step towards obtaining a short-course macrofilaricidal therapy for LF. This study was carried out based on the work by Turner and colleagues, and we followed their published recommendations [15]. Also, to minimize the risk of cross-resistance, we did not use the rifampicin alone arm since rifampicin is a very important drug in the treatment of tuberculosis and widespread usage may pose a substantial risk of cross-resistance with Mycobacterium.

First, it is worth noting that none of the participants recruited into the study tested positive for MF. This is most likely because of the intensive MDA of ivermectin and albendazole by the NTD programme in Ghana. Ivermectin eliminates MFs from an LF infected person’s blood and the MFs usually repopulate after six months. However, due to the biannual MDA conducted in all LF-endemic areas in Ghana, most infected individuals remain amicrofilaraemic. Indeed, previous studies have demonstrated that circulating microfilarial levels do not necessarily correlate with adult worm burden in LF, particularly in endemic settings where MDA is ongoing, as individuals are commonly amicrofilaraemic [30].

Also, the study revealed that the drug regimens were well tolerated, with no serious adverse events reported across treatment arms. Reddish urine discoloration, predominantly observed in the “RIF + ALB-7 Days (TA1)” and “RIF + ALB-14 Days (TA2)” groups, was an expected outcome due to the colour of rifampicin [31]. Moreover, the occurrence of headaches as a minor side effect across all groups, while non-life-threatening, is in line with the general tolerability profile seen in other studies which assessed the macrofilaricidal efficacy of drugs such as doxycycline against LF [32].

### 4.1. Assessment of Treatment Efficacy

All the treatment arms had a reduction in the number of FTS/CFA positive cases at each follow-up compared to the baseline. There was an increasing trend in the rate of reduction in the number of FTS/CFA positive cases over time, with all treatment arms recording the highest reduction at 18-months post-treatment. The results of the Og4C3 TropBio enzyme-linked immunosorbent assay (ELISA) test showed a similar trend to the FTS/CFA results, showing an increasing trend in the rate of Og4C3/CFA seroreversion (change from CFA positive to CFA negative) over time in each treatment arm. Notably, all treatment arms, except the “no treatment” group, had their highest rate of Og4C3/CFA seroreversion at the 18-month follow-up. Furthermore, all the treatment arms had significantly lower antigen levels at the 12- and 18-month follow-up points as compared to the baseline.

Nevertheless, even though all the treatment arms had a decrease in the number of FTS/CFA positive and Og4C3/CFA-positive cases across follow-ups, treatment arm 1 (RIF + ALB-7 Days) consistently showed the highest rate of FTS/CFA seroreversion across all follow ups, with treatment arm 3 (ALB alone-14 Days) recording the lowest rate of seroreversion across all follow-up time points. Also, treatment arm 2 (RIF + ALB for 14 days) recorded a significant (*p* = 0.031) reduction in the number of Og4C3/CFA-positive cases by the 18-month follow-up, where all the baseline Og4C3/CFA-positive cases had seroreverted. However, no statistically significant differences in seroreversion rates were observed between treatment arms at baseline or at any follow-up time point, and CFA levels did not differ significantly between arms at any time point. This lack of difference between groups is most likely attributable to the small sample size. But very importantly, the “RIF + ALB-7 Days” and “RIF + ALB-14 Days” treatment arms were the only groups that achieved significant reduction in antigen levels as early as 4-months post-treatment. In contrast, the “ALB-alone-14 Days” and the “no treatment” arms only started to achieve significant reduction in antigen levels at 12-months post-treatment. This implies that the RIF and ALB combination led to faster clearance of CFA, suggesting accelerated killing of the adult worms from the participants, likely through the anti-*Wolbachia* efficacy that was observed in pre-clinical trials [15].

Since all treatment arms had a reduction in the number of CFA positive cases and significant reductions in CFA levels, our results could be interpreted as, either all the treatment arms were equally effective in clearing CFA from the infected individuals, or the decline in the number of CFA positive cases and antigen units is partly due to natural death of worms in the participants. The latter sounds more plausible, given that even the no treatment arm (who only received the standard MDA dose of ALB + IVM) also had reductions in CFA positive cases as well as significant reduction in antigen units eventually. Both ALB and IVM work against MF, with little or no effect on the adult worm. Albendazole works by disrupting β-tubulin polymerization into microtubules, thereby reducing the number of microtubules; this impairs the parasite’s ability to absorb nutrients, ultimately causing the parasite (MF) to die [22]. For IVM, it works by binding to ligand-gated ion channel receptors, hyperpolarizing glutamate-sensitive channels, and blocking the contractile activity of the excretory/secretory vesicles. Consequently, molecules that could modulate the immune response are not released, leaving MFs undefended in the lymph nodes [11]. Therefore, the albendazole alone treatment and the control (no treatment) arms were not expected to produce significant changes in CFA, which is primarily produced by the adult worm [33]. However, given that our study districts have undertaken MDA for over 15 years [9], it is likely that LF transmission has substantially declined in these areas. Consequently, individuals who remain CFA-positive are likely harbouring older adult worms nearing the end of their natural lifespan of approximately eight years [9,34], and some of these worms may have died naturally. This explains why even the “ALB-alone-14 Days” and the “no treatment” arms also had a significant reduction in antigen units. However, although repeated MDA can eventually lead to worm death, the process is slow and may allow continued transmission, reinfection, and potential resurgence. This underscores the importance of our findings, which demonstrate that the combination of RIF plus ALB results in a much more rapid clearance of CFA, hence preventing reinfections and resurgence.

Rifampicin is an antibiotic and exerts macrofilaricidal effects by targeting and eliminating the *Wolbachia* endosymbionts present within filarial worms [11,15,20,21,34,35]. RIF was found to be over 15 times more potent at killing *Wolbachia* in vivo, compared to doxycycline [20]; hence, the efficacy observed in this study is likely due to rifampicin synergizing with ALB to potently deplete *Wolbachia* as was observed in the pre-clinical trial [15], leading to early adult worm death. The exact mechanisms of the synergy between RIF and ALB in killing *Wolbachia* is unclear. However, it is believed that ALB, through its inhibition of β-tubulin, may disrupt *Wolbachia’s* intracellular movement along microtubules, thereby enhancing the effects of the antibiotic [36]. Accordingly, the earlier clearance of CFA observed among participants receiving the RIF-plus-ALB regimens, compared with those receiving ALB alone or control treatment, provides strong evidence of a synergistic effect, consistent with observations reported by Turner and colleagues in the preclinical trial [15].

The findings from this study imply that treatment duration for LF can potentially be shortened to 7 or 14 days, with the added advantages that RIF can be administered to children, pregnant women, and lactating mothers. The treatment can also be administered in *Loa loa* co-endemic areas without fear of severe adverse reactions from *Loa loa* patients [34]. However, in the absence of an effective macrofilaricide for LF, complete eradication will require a multidimensional approach, including effective vector control, optimized MDA strategies, and the use of modern technologies such as mobile phone-based interactive voice response systems, which have been shown to be effective for LF surveillance [37]. Effective morbidity management strategies, particularly improved hygiene practices, are also critical and have been demonstrated to be beneficial for lymphoedema management [38]. Ultimately, adoption of RIF plus ALB as alternative treatment to complement existing LF control and elimination efforts, especially in an endgame scenario, could substantially accelerate progress toward eliminating LF as a public health problem. Nevertheless, further studies are required to fully evaluate the efficacy and programmatic utility of this combination therapy.

### 4.2. Association Between FTS and Og4C3 TropBio ELISA Tests

The FTS is the preferred diagnostic tool for detecting CFA and for monitoring and evaluation under the GPELF [6,39]. The FTS offers operational simplicity that has significantly enhanced the field implementation of LF surveillance. It eliminates the need for cold-chain storage and provides rapid results from blood samples collected at any time of the day [6,39]. However, the FTS was designed primarily as a qualitative diagnostic tool.

The TropBio Og4C3 ELISA is an alternative antigen detection tool that employs the Og4C3 monoclonal antibody to detect the same AD12 epitope of Wb-CFA as the FTS does [40,41]. Unlike the FTS, this assay provides a quantitative measurement of CFA levels, making it especially valuable for evaluating treatment efficacy by comparing antigen levels before and after drug administration [5,9]. However, its application is restricted to laboratory settings due to the need for specialized equipment and its relatively high cost.

Although the FTS is traditionally considered a qualitative diagnostic tool, the semi-quantitative technique of scoring of the FTS results employed by Chesnais and colleagues [42] gives a valuable assessment of CFA levels by analysing differences in the intensity of test bands produced by the FTS. Chesnais and his colleagues [5] suggest that using FTS for rapid quantification of antigen levels could be a valuable tool for assessing changes in CFA levels post-treatment within communities, without the need for ELISA testing, thereby bridging the gap between the FTS and the TropBio ELISA tests.

In the present study, the comparison between FTS grades/scores and antigen levels measured by the Og4C3 TropBio ELISA revealed a number of important insights. Among the 69 FTS/CFA positive individuals recruited, only 34.8% also tested positive via the Og4C3 TropBio ELISA. While this may initially appear as a discrepancy in diagnostic accuracy, it is important to consider the underlying infection dynamics. A key factor could be the amicrofilaraemic status of the individuals. Previous studies [7] indicated that the Og4C3 ELISA may have reduced sensitivity in individuals with low or undetectable microfilarial densities. Given that antigenaemia can persist in the absence of microfilariae [33], the FTS might still detect CFA even when the TropBio ELISA does not reach its sensitivity threshold for antigen detection.

Despite this baseline discrepancy, a significant association (*p* < 0.05) was observed between the FTS and the Og4C3 TropBio ELISA results at all follow-up time-points, suggesting that both methods are aligned in their ability to track changes in antigenaemia post-treatment. This validates the FTS grading system as an assessable and field-appropriate technique for longitudinal monitoring of treatment impact, especially in resource-limited settings where ELISA testing may not be feasible.

Crucially, the correlation between higher FTS grades/scores and higher Og4C3 antigen units adds another layer of diagnostic relevance to the FTS grading system. The statistical significance (*p* < 0.05) observed between FTS grades/scores and Og4C3 antigen units substantiates the relationship between the two diagnostic indicators. This supports the notion that FTS grading can be a valuable, low-cost alternative to ELISA for community-based CFA assessments, especially when considering large-scale monitoring during MDA campaigns or impact evaluations in endemic settings.

These findings have broader implications for the GPELF and similar control initiatives. The ability to semi-quantitatively monitor CFA levels using FTS offers a rapid, scalable, and operationally feasible method for assessing treatment efficacy in the field. This is especially important during post-MDA surveillance phases, where monitoring residual transmission and verifying the interruption of transmission are key priorities.

A key strength of this study is that it provides the first clinical evidence that a short-course combination of high-dose rifampicin and albendazole accelerates clearance of *W. bancrofti* CFA, thereby translating promising pre-clinical findings into a human LF setting. The observation of good tolerability and a markedly shortened treatment duration, highlights the potential programmatic value of this regimen as a feasible macrofilaricidal intervention. However, the study is limited by the absence of microfilaraemic participants. Since the participants were not microfilaraemic, we were unable to directly evaluate the impact of RIF plus ALB on *Wolbachia*, as assessing adult worms in LF infections in humans is challenging [7,43]. In addition, prolonged prior exposure to MDA in the study districts likely resulted in aging adult worm populations, introducing potential confounding from natural worm death and antigen decline. These factors constrain the generalizability of the findings to high-transmission settings. Consequently, while these findings strongly support the potential of RIF plus ALB as a short-course macrofilaricidal therapy for LF, larger trials in microfilaraemic populations with ongoing transmission are recommended to confirm efficacy and define its role in elimination programmes.

## 5. Conclusions

This clinical trial provides the first concerted human evidence that high-dose rifampicin (35 mg/kg), when combined with albendazole, can significantly accelerate the clearance of CFA in individuals with LF. The combination treatment was safe and well tolerated, with early and sustained reductions in CFA levels observed as early as 4-months post-treatment. While reductions in CFA were also observed in the albendazole-alone and no-treatment arms, likely due to natural worm death in a post-MDA setting, the accelerated antigen clearance in the RIF + ALB arms strongly supports the synergistic effect observed in pre-clinical models, likely through enhanced *Wolbachia* depletion. The rapid antigen clearance implies that treatment duration for LF could potentially be shortened to just 7–14 days. This would mark a major advancement in LF control, as rifampicin is safe for children, pregnant women, and can be used in *Loa loa* co-endemic areas. Moreover, the strong correlation between FTS grading and Og4C3 ELISA results validates the use of FTS as a practical field tool for monitoring treatment efficacy. Nevertheless, further research in microfilaraemic populations and with larger sample sizes is recommended to confirm these findings and to better evaluate the direct impact on *Wolbachia* and the adult worm burden. It is also recommended that a similar study should be undertaken in onchocerciasis patients, where the adult worms are found in encapsulated nodules.

## Figures and Tables

**Table 1:** Safety assessments.

		RIF + ALB-7 Days (TA1)	RIF + ALB-14 Days (TA2)	ALB Alone-14 Days (TA3)	No Treatment/Control (TA4)	Total	*p*-Value
No. of participants		17	18	17	17	69	
No. (%) of participants with AEs		16/17 (94.1)	18/18 (100.0)	4/17 (23.5)	2/17 (11.8)	40/69 (58.0)	<0.001 ^a^
No. of AEs types		6	4	5	3	18	
Frequency of AEs occurrence (%)		22 (40.7)	22 (40.7)	6 (11.1)	4 (7.4)	52	
AEs per affected patients	Mean ± SD	1.375 1.088	1.278 0.461	1.500 1.000	2.000 1.414		0.602 ^b^
	Min–Max	1–5	1–2	1–3	1–3		
	Median	1	1	1	2		
AEs per total patients	Mean ± SD	1.294 1.105	1.278 0.461	0.353 0.786	0.235 0.752		<0.001 ^c^
	Min–Max	0–5	1–2	0–3	0–3		
	Median	1	1	0	0		

TA = Treatment arm; RIF = Rifampicin; ALB = Albendazole; AE = Adverse event; SD = Standard deviation, ^a^ Fisher’s exact test, ^b^ Kruskal–Wallis test, ^c^ Multiple comparisons using Dunn’s post hoc test identified significant differences of *p* < 0.001 between the following pairs of groups: TA1 and TA3, TA1 and TA4, TA2 and TA3, TA2 and TA4.

**Table 2:** Baseline demographic and clinical characteristics of participants enrolled on the study.

Variable	Category	RIF + ALB-7 Days (TA1)	RIF + ALB-14 Days (TA2)	ALB Alone-14 Days (TA3)	No Treatment (TA4)	Total	*p*-Value
Randomised and treated		17	18	17	17	69	0.373 ^a^
Sex	Male, *n* (%)	7 (41.2)	9 (50.0)	11 (64.7)	6 (35.3)	33 (47.8)	
	Female, *n* (%)	10 (58.8)	9 (50.0)	6 (35.3)	11 (64.7)	36 (52.2)	
District	Nabdam, *n* (%)	7 (41.2)	8 (44.4)	7 (41.2)	8 (40.1)	30 (43.5)	0.469 ^a^
	Kassena/Nankana West, *n* (%)	5 (29.4)	7 (38.9)	4 (23.5)	4 (23.5)	20 (29.0)	
	Kassena/Nankana East, *n* (%)	5 (29.4)	3 (16.7)	6 (35.3)	5 (29.4)	19 (27.5)	
Age (years)	Mean (SD)	44.82 (8.66)	45.22 (7.85)	40.35 (6.86)	39.29 (9.32)	42.46 (8.46)	0.080 ^b^
	Min–Max	29–55	26–55	32–55	25–53	25–55	
Weight (kg)	Mean (SD)	60.5 (12.1)	59.6 (8.2)	62.8 (7.7)	58.8 (9.0)	60.4 (9.3)	0.634 ^b^
	Min–Max	46.5–89.0	48.0–76.0	52.0–82.5	48.5–75.0	46.5–89.0	
Years in endemic areas	Mean (SD)	42.82 (9.40)	40.50 (11.00)	38.29 (8.58)	37.06 (9.87)	39.68 (9.80)	0.232 ^c^
	Min–Max	26–55	10–52	20–55	25–53	10–55	
FTS-CFA	Grade 1, *n* (%)	12 (70.6)	12 (66.7)	12 (70.6)	13 (76.5)	49 (71.0)	0.934 ^a^
	Grade 2, *n* (%)	4 (23.5)	6 (33.3)	5 (29.4)	4 (23.5)	19 (27.5)	
	Grade 3, *n* (%)	1 (5.9)	0 (0.0)	0 (0.0)	0 (0.0)	1 (1.4)	
Og4C3 CFA	Positive, *n* (%)	5 (29.4)	6 (33.3)	8 (47.1)	5 (29.4)	24 (34.8)	0.709 ^a^
	Negative, *n* (%)	12 (70.6)	12 (66.7)	9 (52.9)	12 (70.6)	45 (65.2)	
IVM rounds	0 round, *n* (%)	1 (5.9)	0 (0.0)	1 (5.9)	2 (11.8)	4 (5.8)	0.758 ^a^
	1–4 rounds, *n* (%)	8 (47.1)	6 (33.3)	8 (47.1)	6 (35.3)	28 (40.6)	
	≥5 rounds, *n* (%)	8 (47.1)	12 (66.7)	8 (47.1)	9 (52.9)	37 (53.6)	

*n*: Number, CFA: Circulating Filarial Antigen, RIF: Rifampicin, ALB: Albendazole, TA: Treatment Arm, ^a^ Fisher–Freeman–Halton Exact test, ^b^ Analysis of variance (ANOVA) test, ^c^ Kruskal–Wallis test.

**Table 3:** Changes in CFA status within each treatment arm at the various study time-points.

Test	Time-Point	CFA Status	RIF + ALB-7 Days (TA1)	RIF + ALB-14 Days (TA2)	ALB Alone-14 Days (TA3)	No Treatment (TA4)	Total	*p*-Value ^a^
Filariasis Test Strip Test	Baseline	Positive, *n* (%)	17 (100.0)	17 (100.0)	17 (100.0)	17 (100.0)	68 (100.0)	-
	4 months	Positive, *n* (%)	7 (46.7)	11 (68.8)	12 (70.6)	9 (60.0)	39 (61.9)	0.527
		Negative, *n* (%)	8 (53.3)	5 (31.2)	5 (29.4)	6 (40.0)	24 (38.1)	
		Total (*n*)	15	16	17	15	63	
	12 Months	Positive, *n* (%)	10 (66.7)	12 (80.0)	15 (88.2)	10 (76.9)	47 (78.3)	0.445
		Negative, *n* (%)	5 (33.3)	3 (20.0)	2 (11.8)	3 (23.1)	13 (21.7)	
		Total (*n*)	15	15	17	13	60	
	18 Months	Positive, *n* (%)	6 (42.9)	7 (46.7)	10 (66.7)	6 {46.2)	29 (50.9)	0.412
		Negative, *n* (%)	8 (57.1)	8 (53.3)	5 (33.3)	7 (53.8)	28 (49.1)	
		Total (*n*)	14	15	15	13	57	
Og4C3 TropBio ELISA Test	Baseline	Positive, *n* (%)	5 (29.4)	6 (35.3)	8 (47.1)	5 (29.4)	24 (35.3)	0.709
		Negative, *n* (%)	12 (70.6)	11 (64.7)	9 (52.9)	12 (70.6)	45 (64.7)	
		Total (*n*)	17	17	17	17	68	
	4 months	Positive, *n* (%)	3 (20.0) ^b^	4 (25.0) ^b^	7 (41.2) ^b^	6 (40.0) ^b^	20 (31.7)	0.476
		Negative, *n* (%)	12 (80.0)	12 (75.0)	10 (58.8)	9 (60.0)	43 (68.3)	
		Total (*n*)	15	16	17	15	63	
	12 Months	Positive, *n* (%)	4 (26.7) ^b^	4 (26.7) ^b^	3 (17.6) ^b^	3 (23.1) ^b^	14 (23.3)	0.976
		Negative, *n* (%)	11 (73.3)	11 (73.3)	14 (82.4)	10 (76.9)	46 (76.7)	
		Total (*n*)	15	15	17	13	60	
	18 Months	Positive, *n* (%)	3 (21.4) ^b^	0 (0.0) ^c^	1 (6.7) ^b^	3 (23.1) ^b^	7 (12.3)	0.238
		Negative, *n* (%)	11 (78.6)	15 (100.0)	14 (93.3)	10 (76.9)	50 (87.7)	
		Total (*n*)	14	15	15	13	57	

^a^ Fisher–Freeman–Halton Exact test ^b,c^: Compared to baseline (McNemar–Bowker test__ ^b^ *p* > 0.05; ^c^ *p* = 0.031), ELISA: Enzyme-Linked Immunosorbent Assay, RIF: Rifampicin, ALB: Albendazole, CFA: Circulating Filarial Antigen, TA: Treatment Arm. Only participants who were treated per protocol (PP) and had taken IVM at 6 months were included in analysis for 12- and 18-month follow ups. Also, statistical comparisons between baseline and follow ups were made among the same participants.

**Table 4:** ITT comparison of FTS/CFA and Og4C3 CFA results across study time-points.

Time-Point		Og4C3 CFA Status, *n* (%)			*p*-Value (Fisher’s Exact Test)
	FTS/CFA Status	Negative	Positive	Total	
Baseline	Positive	45 (65.2)	24 (34.8)	69 (100.0)	-
4 Months	Negative	22 (88.0)	3 (12.0)	25 (100.0)	0.012
	Positive	22 (56.4)	17 (43.6)	39 (100.0)	
	Total	44	20	64	
12 Months	Negative	15 (100.0)	0 (0.0)	15 (100.0)	0.028
	Positive	35 (72.4)	14 (28.6)	49 (100.0)	
	Total	50	14	64	
18 Months	Negative	31 (100.0)	0 (0.0)	31 (100.0)	0.004
	Positive	22 (75.9)	7 (24.1)	29 (100.0)	
	Total	53	7	60	

ITT—Intention-to-Treat, FTS—Filariasis Test Strip, CFA—Circulating Filarial Antigen.

**Figure A6:**
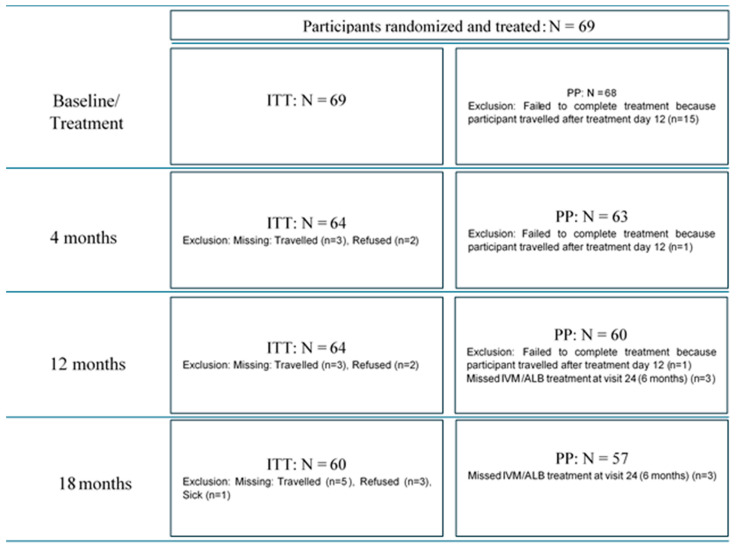
Flowchart of the included participants in the ITT and PP analysis sets. Abbreviations: N/*n* = Number, ITT = Intention-to-Treat, PP = Per-Protocol, IVM = Ivermectin, ALB = Albendazole.

## Data Availability

The data that support, the study protocol, the statistical analysis plan, and other documents of this study are available from the corresponding author upon request.

## Abbreviations

The following abbreviations are used in this manuscript:

AE	Adverse Events
ALB	Albendazole
ALT	Alanine Aminotransferase
ANOVA	Analysis of variance
AST	Aspartate Aminotransferase
CFA	Circulating Filarial Antigen
CHRPE	Committee on Human Research Publication and Ethics
DEC	Diethylcarbamazine
DOT	Directly Observed Treatment
DSMB	Data Safety and Monitoring Board
EDCTP	European and Developing Countries Clinical Trials Partnership
EDTA	Ethylenediaminetetraacetic acid
eGFR	estimated Glomerular Filtration Rate
ELISA	Enzyme-Linked Immunosorbent Assay
ERC	Ethics Review Committee
FDA	Food and Drugs Authority
FTS	Filariasis Test Strip
GCP	Good Clinical Practice
GGT	Gamma-Glutamyl Transferase
GHS	Ghana Health Service
GPELF	Global Programme to Eliminate Lymphatic Filariasis
GWAC	German-West African Center for Global Health and Pandemic Prevention
HRP	Horseradish Peroxidase
ID	Identification
ITT	Intention-to-treat
IVM	Ivermectin
KCCR	Kumasi Centre for Collaborative Research in Tropical Medicine
KNUST	Kwame Nkrumah University of Science and Technology
LE	Lymphoedema
LF	Lymphatic Filariasis
MDA	Mass Drug Administration
MF	Microfilariae
MMDP	Morbidity Management and Disability Prevention
PP	Per Protocol
REDCap	Research Electronic Data Capture
RIF	Rifampicin
SAEs	Serious Adverse Events
SD	Standard Deviation
TA	Treatment Arm
TMB	Tetramethylbenzidine
TSC	Trial Steering Committee
WHO	World Health Organisation

## Appendix A

**Table A1:** Adverse event assessment, detailed.

Adverse Events			RIF + ALB-7 Days (TA1), N (%)	RIF + ALB-14 Days (TA2), N (%)	ALB Alone-14 Days (TA3), N (%)	No Treatment/Control (TA4), N (%)	*p*-Value ^a^
Reddish coloured urine	No. of Participants with AEs		16/17 (94.1)	18/18 (100.0)	1/17 (5.9)	0	0.000
	Grade of AE *	Mild (Grade1)	16 (100.0)	18 (100.0)	1 (100.0)		
	Related to treatment	Suspected	16 (100.0)	17 (94.4)	1 (100.0)		
		Not suspected	0	1 (5.6)	0		
Headache	No. of AEs		2 (11.8)	1 (5.6)	2 (11.8)	2 (11.8)	0.866
	Grade of AE *	Mild (Grade1)	2 (100.0)	1 (100.0)	0	0	
		Moderate (Grade 2)	0	0	2 (100.0)	2 (100.0)	
	Related to treatment	Suspected	2 (100.0)	1 (100.0)	2 (100.0)	0	
		Not suspected	0	0	0	2 (100.0)	
Malaria	No. of AEs		0	0	0	1 (5.9)	0.739
	Grade of AE *	Moderate (Grade 2)				1 (100.0)	
	Related to treatment	Not suspected				1 (100.0)	
Body itch	No. of AEs		0	2 (11.1)	0	0	0.239
	Grade of AE *	Mild (Grade 1)		2 (100.0)			
	Related to treatment	Suspected		1 (50.0)			
		Not suspected		1 (50.0)			
Others	No. of AEs		2 (11.8)	1 (5.6)	2 (11.8)	1 (5.9)	0.850
	Grade of AE *	Mild (Grade 1)		1 (100.0)	2 (100.0)	0	
		Moderate (Grade 2)	0	0	0	1 (100.0)	
	Related to treatment	Suspected	2 (50.0)	1 (100.0)	0	0	
		Not suspected	0	0	2 (100.0)	1 (100.0)	

N = Number; TA = Treatment arm; RIF = Rifampicin; ALB = Albendazole; AE = Adverse event. Fisher’s exact test. * Grading was always “Mild (Grade 1)”, “Moderate (Grade 2)”, “Severe (Grade 3)”. Only the grades which occurred are shown. ^a^ Fisher’s exact test.

**Table A2:** Changes in FTS-CFA status within each treatment arm across the various study time-points.

Test	Time-Point	FTS-CFA Status	RIF + ALB-7 Days (TA1)	RIF + ALB-14 Days (TA2)	ALB Alone-14 Days (TA3)	No Treatment (TA4)	Total	*p*-Value ^a^
FTS-CFA	Baseline	Positive, *n* (%)	17 (100.0)	18 (100.0)	17 (100.0)	17 (100.0)	69 (100.0)	-
	4months	Positive, *n* (%)	7 (46.7)	11 (64.7)	12 (70.6)	9 (60.0)	39 (60.9)	0.599
		Negative, *n* (%)	8 (53.3)	6 (35.3)	5 (29.4)	6 (40.0)	25 (39.1)	
		Total (*n*)	15	17	17	15	64	
	12 Months	Positive, *n* (%)	11 (64.7)	12 (75.0)	15 (88.2)	11 (78.6)	49 (76.6)	0.454
		Negative, *n* (%)	6 (35.3)	4 (25.0)	2 (11.8)	3 (21.4)	15 (23.4)	
		Total (*n*)	17	16	17	14	64	
	18 Months	Positive, *n* (%)	6 (37.5)	7 (46.7)	10 (66.7)	6 (42.9)	29 (48.3)	0.412
		Negative, *n* (%)	10 (62.5)	8 (53.3)	5 (33.3)	8 (57.1)	31 (51.7)	
		Total (*n*)	16	15	15	14	60	
Og4C3 CFA status	Baseline	Positive, *n* (%)	5 (29.4)	6 (33.3)	8 (47.1)	5 (29.4)	24 (34.8)	0.709
		Negative, *n* (%)	12 (70.6)	12 (66.7)	9 (52.9)	12 (70.6)	45 (65.2)	
		Total (*n*)	17	18	17	17	69	
	4months	Positive, *n* (%)	3 (20.0) ^b^	4 (23.5) ^b^	7 (41.2) ^b^	6 (40.0) ^b^	20 (31.3)	0.476
		Negative, *n* (%)	12 (80.0)	13 (76.5)	10 (58.8)	9 (60.0)	44 (68.7)	
		Total (*n*)	15	17	17	15	64	
	12 Months	Positive, *n* (%)	4 (23.5) ^b^	4 (25.0) ^b^	3 (17.7) ^b^	3 (21.4) ^b^	14 (21.9)	0.976
		Negative, *n* (%)	13 (76.5)	12 (75.0)	14 (82.3)	11 (78.6)	50 (78.1)	
		Total (*n*)	17	16	17	14	64	
	18 Months	Positive, *n* (%)	3 (18.8) ^b^	0 (0.0) ^c^	1 (6.7) ^b^	3 (21.4) ^b^	7 (11.7)	0.238
		Negative, *n* (%)	13 (81.2)	15 (100.0)	14 (93.3)	11 (78.6)	53 (88.3)	
		Total (*n*)	16	15	15	14	60	

^a^ Fisher–Freeman–Halton Exact test, ^b,c^: Compared to baseline (McNemer–Bowker test__ ^b^ *p* > 0.05; ^c^ *p* = 0.031), RIF: Rifampicin, ALB: Albendazole, TA: Treatment Arm, *n*: Number.
